# Chromosomes of Theridiidae spiders (Entelegynae): Interspecific karyotype diversity in *Argyrodes* and diploid number intraspecific variability in *Nesticodes rufipes*

**DOI:** 10.1590/S1415-47572010005000076

**Published:** 2010-12-01

**Authors:** Leila Miguel Stavale, Marielle Cristina Schneider, Douglas Araujo, Antonio Domingos Brescovit, Doralice Maria Cella

**Affiliations:** 1 Departamento de Biologia, Universidade Estadual Paulista Júlio de Mesquita Filho, Rio Claro, SPBrazil; 2 Departamento de Ciências Biológicas, Universidade Federal de São Paulo, Diadema, SPBrazil; 3 Unidade Universitária de Mundo Novo, Universidade Estadual de Mato Grosso do Sul, Mundo Novo, MSBrazil; 4 Laboratório de Artrópodes, Instituto Butantan, São Paulo, SPBrazil

**Keywords:** chromosome morphology, cytogenetic, meiosis, nucleolar organizer region, sex chromosome system

## Abstract

Theridiidae is a derived family within the Araneoidea clade. In contrast to closely related groups, the 2n(male) = 20+X_1_ X _2_ with acro/telocentric chromosomes is the most widespread karyotype among the theridiid spiders. In this work, the cytogenetic analysis of *Argyrodes elevatus* revealed original chromosome features different from those previously registered for Theridiidae, including the presence of 2n(male) = 20+X with meta/submetacentric chromosomes. Most individuals of *Nesticodes rufipes* showed family conserved karyotype characteristics. However, one individual had a 2n(male) = 24 due to the presence of an extra chromosome pair, which exhibited regular behavior and reductional segregation during meiosis. After silver staining, mitotic cells exhibited NORs localized on the terminal regions of the short arms of pairs 2, 3, and 4 of *A. elevatus* and on the terminal regions of long arms of pair 4 of *N. rufipes*. The comparative analysis with data from phylogenetically related species allowed the clarification of the origin of the interspecific and intraspecific chromosome variability observed in *Argyrodes* and in *N. rufipes*, respectively.

## Introduction

Theridiidae is among the largest families of the order Araneae, including 2.297 species subdivided into 112 genera (Platnick, 2010). The extreme diversity of foraging and lifestyle strategies, which range from solitary webless species to social spiders with maternal care, was certainly the factor that contributed to the diversification of the theridiids (Agnarsson, 2004; Arnedo *et al.*, 2004). Within this family, the cosmopolitan species of the genus *Argyrodes* are famous for their kleptoparasite behavior. The kleptoparasite spiders invade the webs of unrelated and usually larger species to steal food or silk (Whitehouse *et al.*, 2002; Agnarsson, 2004). Among the theridiids, most species of the genus *Nesticodes* have a synanthropic behavior, being frequently found in association with human habitations where it is easy to obtain food (Cushing and LeBeck, 1994; Rossi and Godoy, 2006).

The family Theridiidae belongs to the Araneoidea group, which includes almost one third of all taxonomically described spiders (Platnick, 2010). In contrast to the five other families of Araneoidea subjected to cytogenetic analyses (Araneidae, Linyphiidae, Nephilidae, Nesticidae, and Tetragnathidae), which exhibited a predominance of 2n(male) = 24 = 22+X_1_X_2_, 23 Theridiidae species showed a 2n(male) = 22, including a sex chromosome system of the X_1_X_2_ type and acro/telocentric chromosomes. Among eight other theridiids, the 2n(male) = 22+X_1_X_2_ was observed in *Argyrodes gazingensis*, *Chrysso scintillans*, and *Parasteatoda tepidariorum*, and chromosome numbers ranging from 2n(female) = 16 to 2n(female) = 28 were reported for five species of *Latrodectus*. The chromosome morphology was only described for three of these eight species, which exhibited acro/telocentric chromosomes (reviewed in Araujo *et al.*, 2010).

Considering that only one Brazilian species of Theridiidae has been cytogenetically studied to date and the discrepant chromosome numbers found in this family in relation to other araneoids, this work aimed to characterize the mitotic and meiotic chromosomes of *Argyrodes elevatus* Taczanowski, 1873 and *Nesticodes rufipes* (Lucas, 1846). The chromosomal analyses were performed in gonadal and embryonic cells after standard staining with Giemsa and silver impregnation. The results were compared with those of related species to establish the main trends of chromosome evolution within Theridiidae.

## Material and Methods

The sample of 58 individuals analyzed in this work comprised: *A. elevatus* - 13 adult males and 13 embryos (eight males and five females) from Rio Claro (22°23' S, 47°32' W), São Paulo (SP), Brazil, and 10 embryos (four males and six females) from Tupã (21°56' S, 50°30' W), SP; *N. rufipes* - 12 adults (five males and seven females) and four male embryos from Rio Claro, SP, and one adult male and five embryos (two males and three females) from Viçosa (20°45' S, 44°52' W), Minas Gerais, Brazil. The sex of the embryos was determined according to their karyotype. The adult specimens were deposited in the collection of the Laboratório de Artrópodes, Instituto Butantan (IBSP), São Paulo, SP. The chromosome preparations were obtained from adult gonads and from embryos, according to the methodology described by Araujo *et al.* (2008). Chromosome spreads were stained with Giemsa (3% of commercial Giemsa and 3% of phosphate buffer pH 6.8, in distilled water) for 15 min, followed by silver nitrate impregnation (Howell and Black, 1980) to reveal the nucleolar organizer regions (NORs). The chromosome analysis was performed under an Olympus BX51 light microscope and the images of the mitotic and meiotic cells were captured using the DP Controller software. The nomenclature for chromosome morphology followed Levan *et al.* (1964).

## Results

**Figure 1:**
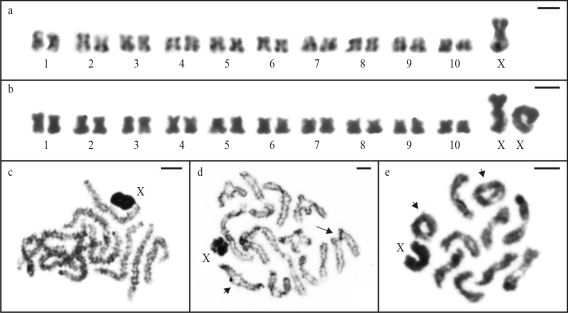
Mitotic and meiotic cells of *Argyrodes elevatus* stained with Giemsa. Karyotypes of male (a) and female (b) embryos, with 2n = 20+X and 2n = 20+XX, respectively. Observe the large size of the X chromosome. (c) Pachytene, (d) diplotene and (e) diakinesis, with 10II+X, exhibiting bivalents with interstitial (large arrow) or terminal (small arrow) chiasmata. Note the bivalents with two terminal chiasmata in (e). Scale bar = 5 μm.

Mitotic metaphase cells of *A. elevatus* showed a diploid number 2n = 21 for males and 2n = 22 for females with a sex chromosome system of the X/XX type and meta/submetacentric chromosomes (Figure 1a,b). The autosome pairs gradually decreased in size and the X chromosome was extremely large. In males, pachytene cells presented ten totally synapsed autosomal bivalents plus one highly condensed and strongly stained chromosome, which was identified as the univalent X chromosome (Figure 1c). Diplotene and diakinesis nuclei showed up to three autosomal bivalents with two terminal chiasmata. The other bivalents presented only one interstitial or terminal chiasma (Figure 1d,e). In these late prophase I stages, the X chromosome also revealed a higher degree of condensation in relation to the autosomes.

**Figure 2:**
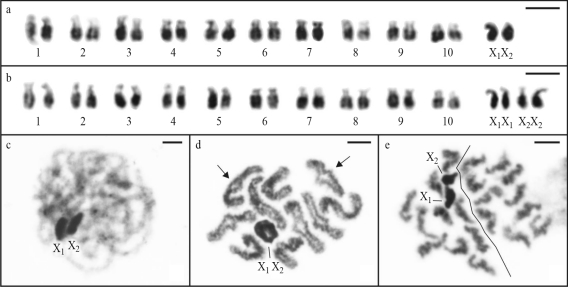
Mitotic and meiotic cells of *Nesticodes rufipes* stained with Giemsa. Karyotypes of male (a) and female (b) embryos, with 2n = 20+X_1_X_2_ and 2n = 20+X_1_X_1_X_2_X_2_, respectively. (c) Pachytene, (d) diplotene, 10II+X_1_X_2_, showing autosomal bivalents with one terminal chiasma (arrow). (e) Metaphase II nuclei, with n = 10+X_1_X_2_ and n = 10. Scale bar = 5 μm.

The karyotypes of 12 adults and 9 embryos of *N. rufipes* had a diploid number 2n = 22 in males and 2n = 24 in females, which were consistent with a sex chromosome system of the X_1_X_2_/X_1_X_1_X_2_X_2_ type (Figure 2a-b). In this species, all chromosomes were acrocentric with gradually decreasing sizes. The medium-sized sex chromosomes were slightly more condensed than the autosomes. Male prophase I cells revealed two highly condensed stained blocks disposed side by side, confirming the X_1_X_2_ sex chromosome system in this species (Figure 2c). Diplotene nuclei had the meiotic formula 10II+X_1_X_2_ and all autosomal bivalents showed only one interstitial or terminal chiasma (Figure 2d). Metaphase II cells exhibited n = 10+X_1_X_2_ and n = 10 (Figure 2e).

**Figure 3:**
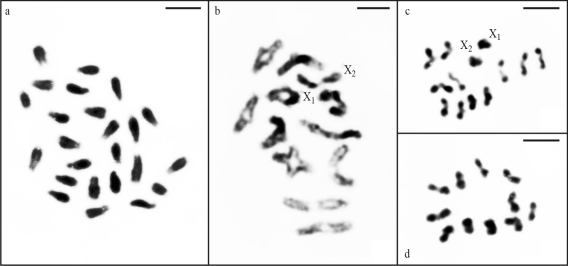
Mitotic and meiotic testicular cells of *Nesticodes rufipes* stained with Giemsa. (a) Metaphase with 2n = 24. (b) Diplotene, revealing 11 autosomal bivalents plus X_1_ and X_2_. Metaphase II nuclei, with n = 11+X_1_X_2_ (c) and n = 11 (d). Scale bar = 5 μm.

Mitotic cells of one adult male of *N. rufipes* from Viçosa revealed 2n = 24 with acrocentric chromosomes, differing from all other males analyzed (Figure 3a). In these cells, the sex chromosomes did not exhibit differential cytological features that allowed their identification. Male diplotene and diakinesis nuclei showed 11 autosomal bivalents and two sex chromosomes arranged side by side or in close proximity (Figure 3b). Metaphase II cells revealed two kinds of haploid sets: n = 11+X_1_X_2_ and n = 11 (Figure 3c,d).

**Figure 4:**
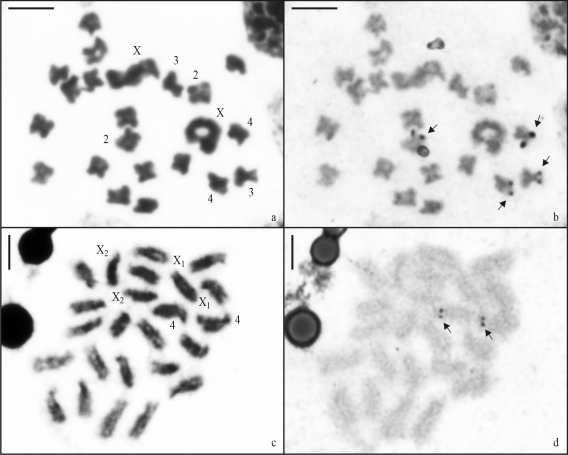
Mitotic cells of *Argyrodes elevatus* (a-b) and *Nesticodes rufipes* (c-d) after Giemsa staining (a and c) and silver nitrate impregnation (b and d). (a) Female, 2n = 20+XX; (b) the same cell with NORs (arrow) on the terminal regions of the short arms of pairs 2, 3, and 4. (c) Female, 2n = 20+X_1_X_1_X_2_X_2_; (d) The same nucleus with NORs (arrow) on the terminal regions of the long arms of pair 4. Scale bar = 5 μm.

After silver impregnation, mitotic metaphase cells of *A. elevatus* revealed NORs on the terminal regions of the short arms of pairs 2, 3, and 4. The number of active NORs varied from two to six per cell (Figure 4a,b). In *N. rufipes*, the NORs were localized on the terminal regions of the long arms of pair 4 (Figure 4c,d).

## Discussion

The chromosomal characteristics observed in *A. elevatus* were extremely discrepant from those described for 30 species of Theridiidae previously studied, including five representatives of the genus *Argyrodes* (see Araujo *et al.*, 2010). Thus, this is the first record of the karyotype formula 2n(male) = 21 = 20+X with biarmed chromosomes for a theridiid spider. In contrast, the karyotype with 2n(male) = 22, the X_1_X_2_ sex chromosome system and acrocentric chromosomes found in most *N. rufipes* studied herein is similar to that predominantly found in the family.

Theridiidae and its sister-group Nesticidae constitute the theridioids, a derived branch within the Araneoidea clade (Griswold *et al.*, 1998; Agnarsson, 2004). The karyotype 2n(male) = 22+X_1_X_2_ with acro/telocentric chromosomes is highly conserved among the Araneoidea spiders, considering that, with the exception of Theridiidae, it was observed in approximately 80% of the species belonging to five different families (Araujo D, PhD Thesis, Instituto de Biociências de Rio Claro, UNESP, São Paulo, 2007). Nevertheless, among the theridiids, the karyotype with 2n(male) = 20+X_1_X_2_, and acro/telocentric chromosomes is the most widespread and was already observed in species of all subfamilies already investigated (Araujo *et al.*, 2010). It appears thus that the main trend of chromosome evolution within Araneoidea was the reduction of the diploid number with the conservation of the sex chromosome system. In Theridiidae, diploid numbers higher than 2n(male) = 22, such as the 2n(male) = 24 observed in one species of *Argyrodes*, *Chrysso*, and in some individuals of *Parasteatoda tepidariorum* (Montgomery, 1907; Kageyama and Seto, 1979; Datta and Chatterjee, 1983), 2n(female) = 28 and 2n(female) = 26 found in species of *Latrodectus*, as well as diploid numbers lower than 2n(male) = 22, such as 2n(female) = 16 and 2n(female) = 18 also reported in *Latrodectus* (Araujo *et al.*, 2010) and the 2n(male) = 21 of *A. elevatus*, probably correspond to a derived condition originated from the 2n(male) = 22.

Considering that the 2n(male) = 20+X_1_X_2_ could represent a basal condition for Theridiidae, the karyotype with 2n(male) = 20+X of *A. elevatus* would not have originated through changes in the number of autosomal pairs, but rather by a change in the sex chromosome system and in the morphology of the autosomes. The X type sex chromosome system probably derived from the X_1_X_2_ system after a Robertsonian translocation between the acro/telocentric X_1_ and X_2_ chromosomes. This hypothesis is reinforced by the fact that the X chromosome of *A. elevatus* is a large biarmed element. Additionally, the morphological change of all autosomes from acro/telocentric to meta/submetacentric probably resulted from pericentric inversions. An alternative mechanism would be the addition of constitutive heterochromatin.

In Entelegynae, a derived lineage within the suborder Araneomorphae, the X sex chromosome system also seems to have evolved secondarily from an X_1_X_2_ system with independent origins in different species or families (Král *et al.*, 2006; Araujo D, PhD Thesis, Instituto de Biociências de Rio Claro, UNESP, São Paulo, 2007). The presence of metacentric or submetacentric chromosomes such as observed in *A. elevatus* is extremely sporadic among the entelegyne spiders. In this group, the change from acro/telocentric to meta/submetacentric chromosomes has been generally attributed to centric fusions involving all chromosomes of the complement. This proposition of chromosome evolution via “all or nothing” fusion (Rowell, 1990) has been corroborated by the fact that the species with a predominance of biarmed chromosomes have a lower diploid number than those with acro/telocentric chromosomes (Mittal, 1966; Rowell, 1988, 1990, 1991; Amalin *et al.*, 1993). Nevertheless, the mechanism of pericentric inversions observed in *A.elevatus* may also be responsible for the dramatic karyotype evolution of the Entelegynae.

The karyotype of most *N. rufipes* specimens was similar to the one considered conserved for Theridiidae. However, the mitotic and meiotic cells of one adult male showed two extra chromosomes. The diploid number variation in this individual was certainly not due to chromosome fission as the chromosomes were of uniform size, *i.e.*, no autosomal pair exhibited a remarkable difference in size that could result from fission. Slight intraspecific variations in chromosome numbers have been frequently reported for spiders (Araujo D, PhD Thesis, Instituto de Biociências de Rio Claro, UNESP, São Paulo, 2007); but no further explanation for these variation has been put forward as yet. The 2n(male) = 24 in *N. rufipes* probably resulted from the presence of one additional autosome pair, considering that in diplotene and diakinesis nuclei, 11 instead of ten autosomal bivalents were invariably observed. Moreover, the metaphase II cells always showed the haploid sets n = 11+X_1_X_2_ and n = 11. Taking into account that the extra chromosome pair was seen in all cells of this individual, it is possible to infer that it originated by a meiotic non-disjunction during the formation of the maternal or paternal gametes. Alternatively, considering the haploid numbers verified in the metaphase II nuclei, this extra chromosome pair could correspond to B chromosomes with a regular meiotic behavior following a Mendelian transmission rate.

There are no previous records on the NOR distribution pattern in theridiid spiders in the literature. Nucleolar organizer regions on autosomes, such as those observed in *A. elevatus* and *N. rufipes*, are also the most frequent condition in Entelegynae, in which they were observed in all investigated species (Wise 1983; Barrion *et al.*, 1989; Araujo *et al.*, 2005; Rodriguez-Gil *et al.*, 2007). Although the two theridiids exhibited great karyotypic differences, it is interesting to note that both species showed NORs on the terminal regions of one medium-sized autosomal pair (pair 4). The analysis of a larger number of Theridiidae spiders could reveal if NORs on medium-sized autosomal elements are a shared feature of the family. The increase of NORs numbers in *A. elevatus* may be a derived condition originated by duplications followed by translocations. In Oxyopidae spiders (Entelegynae), we also observed a relationship between a large number of NORs and an extremely derived karyotype.
